# How to demonstrate the prognostic impact of interdialytic home blood pressure variability in dialytic patients

**DOI:** 10.1002/clc.24279

**Published:** 2024-05-22

**Authors:** Nanako Marubayashi, Kota Kakeshita, Teruhiko Imamura

**Affiliations:** ^1^ Second Department of Internal Medicine University of Toyama Toyama Japan

To the Editor,

Dong et al.^1^ investigated the prognostic impact of interdialytic home blood pressure variability (BPV) in individuals undergoing maintenance hemodialysis. They demonstrated that an incremental interdialytic home BPV was associated with mortality in this cohort. Several concerns have been raised.

In the authors' study, home blood pressure was recorded four times a day for a week on a nondialysis day and their variability was calculated.^1^ Another concern is the prognostic impact of intraday BPV during a day or interday BPV during several days.

Blood pressure recorded either predialysis or postdialysis displays a U‐shaped curve with mortality among dialysis patients.^2^ In the authors' study, they did not adjust for systolic blood pressure to evaluate the prognostic impact of BPV.^1^ Patients with low systolic blood pressure may have incremental mortality even though they have high BPV.

Incremental BPV was associated with not only cardiac death but also all‐cause death.^1^ Could the authors state detailed causes of noncardiac death? Also, an incremental number of dialysis patients are found to have severe aortic stenosis, which can be treated by transcatheter aortic valve replacement, if applicable.^3^ The prevalence of such diseases may increase as incremental BPV. Did the authors' patients have any valvular diseases?

Accurate measurement of blood pressure is challenging in patients with atrial fibrillation. However, the prevalence of atrial fibrillation is high in dialysis patients.^4^ Atrial fibrillation is associated with mortality and morbidity. The indication of anti‐coagulants in dialysis patients with atrial fibrillation is controversial. Moreover, percutaneous left atrial appendage closure in this cohort has recently become available with acceptable feasibility.^5^ Do the authors have any individuals with atrial fibrillation?

## Data Availability

Data sharing is not applicable to this article as no datasets were generated or analyzed during the current study.
